# An In Silico Deep Learning Approach to Multi-Epitope Vaccine Design: A Hepatitis E Virus Case Study

**DOI:** 10.3390/vaccines11030710

**Published:** 2023-03-22

**Authors:** Aqsa Ikram, Badr Alzahrani, Tahreem Zaheer, Sobia Sattar, Sidra Rasheed, Muhammad Aurangzeb, Yasmeen Ishaq

**Affiliations:** 1 Institute of Molecular Biology and Biotechnology (IMBB), University of Lahore (UOL), Lahore 54000, Pakistan; 2 Department of Clinical Laboratory Sciences, College of Applied Medical Sciences, Jouf University, Sakaka 72388, Saudi Arabia; 3 Department of Biological Physics, Eötvös Loránd University, Pázmány Péter Sétány 1/A, 1117 Budapest, Hungary

**Keywords:** HEV, immunoinformatic, multi-epitope, proposed, vaccine

## Abstract

Hepatitis E Virus (HEV) is a major cause of acute and chronic hepatitis. The severity of HEV infection increases manyfold in pregnant women and immunocompromised patients. Despite the extensive research on HEV in the last few decades, there is no widely available vaccine yet. In the current study, immunoinformatic analyses were applied to predict a multi-epitope vaccine candidate against HEV. From the ORF2 region, 41 conserved and immunogenic epitopes were prioritized. These epitopes were further analyzed for their probable antigenic and non-allergenic combinations with several linkers. The stability of the vaccine construct was confirmed by molecular dynamic simulations. The vaccine construct is potentially antigenic and docking analysis revealed stable interactions with TLR3. These results suggest that the proposed vaccine can efficiently stimulate both cellular and humoral immune responses. However, further studies are needed to determine the immunogenicity of the vaccine construct.

## 1. Introduction

Hepatitis E virus (HEV) is a common cause of acute hepatitis and is mainly transmitted through contaminated food and drinking water. It has been estimated that almost 20 million HEV cases, 3.4 million symptomatic infections, and 70,000 deaths occur annually in the world [1].Endemic HEV is a serious threat to life and productivity in the developing regions of the world, but a persistently increasing number of HEV infections have also been reported in developed countries. Therefore, HEV is also an emerging public health threat in the developed world. HEV has expanded host ranges; its cross-species transmission has recognized HEV as a zoonotic disease and raised a serious health concern for the human population [2]. Moreover, immunocompromised persons, pregnant women, and those diagnosed with pre-existing chronic liver diseases are at particular risk of worse outcomes. The above-mentioned facts highlight the importance of HEV as a global health problem [1].

Although scientists are constantly working on developing an HEV vaccine during the last several decades, an effective HEV vaccine, either inactivated or live-attenuated, has not been produced due to lack of effective cell culture systems for robust HEV replication [3]. Fortunately, capsid protein of all major HEV genotypes have more than 85% sequence identity. Therefore, HEV vaccine production relies upon recombinant HEV capsid proteins as subunit or antigenic region vaccines. Interestingly, a subunit-based HEV vaccine has been successfully developed and approved in China. However, it is not commercially available elsewhere in the world. Another HEV vaccine named recombinant HEV (rHEV) was produced using GlaxoSmithKline funding, but this vaccine has not been approved for commercial use due to unknown reasons [4]. It is, therefore, anticipated that an effective HEV vaccine is urgently needed to control HEV infection.

**Figure 1:**
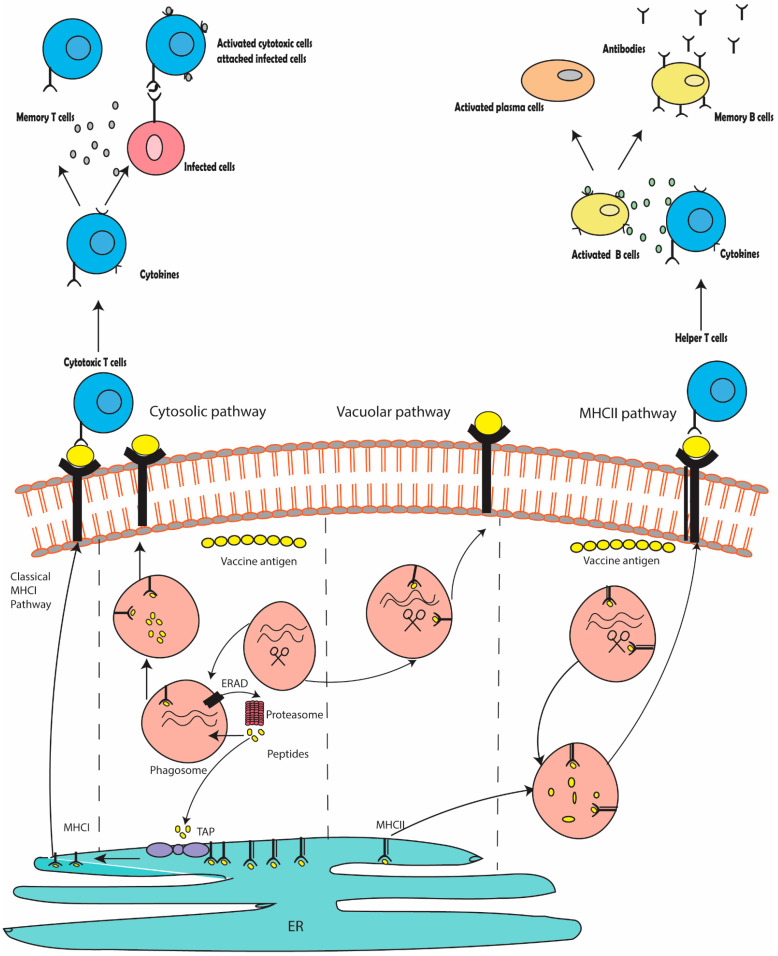
The molecular mechanism of immune responses stimulated by epitope-based vaccine candidate.

Immunoinformatic analysis has played an important role in determining the immune response of various antigenic regions by using computational techniques and resources. This allows the selection of epitopes from the viral and bacterial genomes. The ideal epitopes can be taken as potential vaccine candidates to trigger effective immune responses in the hosts. The immunoinformatic-based multi-epitope vaccine candidate can stimulate both cellular and humoral immunity [5]. Targeted delivery of vaccine antigens to antigen-presenting cells (APCs) is a major goal of immunoinformatic-based vaccines [6]. This enhances vaccine exhibition and is an effective strategy for making more efficient vaccines. Induction of robust T cell responses is required to increase relevance for vaccine efficacy (Figure 1) [7].

Previously, numerous immunoinformatic methods have been used to develop vaccines against various pathogens. The vaccine produced by conventional methods can increase the potential for allergic reactions. However, the epitope-based vaccine might be able to overcome these limitations. In addition, this method is rapid, easy, and cost-effective when compared with other vaccine production methods. Multimeric-001 (M-001) is an example of an epitope-based vaccine that induced humoral and cellular immune responses and is under clinical trials [8] FLU-v is a multi-epitope vaccine identified in silico analysis that generates CD8^+^ T cell response in mice and human cells. Flunisyn™ or FP-01.1 is a chemically synthesized multi-epitope vaccine that also produces CD4^+^ and CD8^+^ T cell responses in mice, when compared with native peptides [9]. These studies demonstrate that immunoinformatic approaches in combination with experimental validation help to identify antigenic regions with better accuracy, thus playing a contributing role in the development of an epitope-based vaccine.

An ideal HEV multi-epitope vaccine should stimulate effective T cell (CD4^+^, CD8^+^) and B cell responses. The HEV whole genome is composed of three open reading frames (ORFs). Among them, ORF2 encodes the major structural protein and has the highest sequence identity. Therefore, HEV capsid protein (ORF2) is the main target for neutralizing antibodies, and most of the experimental vaccines against hepatitis E are based on this antigen [10]. This study was designed to develop a universal vaccine candidate (Vu) against the major HEV genotypes (GT1, GT3, and GT4) based on the ORF2 region, using a large-scale immunoinformatic analysis. Identified epitopes were prioritized to build vaccine constructs that were composed of conserved B cell, T cell, and IFNγ epitopes. All these epitopes were in the experimentally-validated antigenic regions, confirming the authenticity and efficacy of the designed vaccine constructs. Furthermore, the vaccine construct has been designed by linking predicted and prioritized epitopes with linkers, along with an adjuvant β-defensin (45 amino acid; aa) added at the N-terminal end of the vaccine construct. The tertiary structure of the vaccine candidate was predicted, validated, and checked for its molecular interactions with Toll-like receptor 3 (TLR3). 

## 2. Materials and Methods

**Figure 2:**
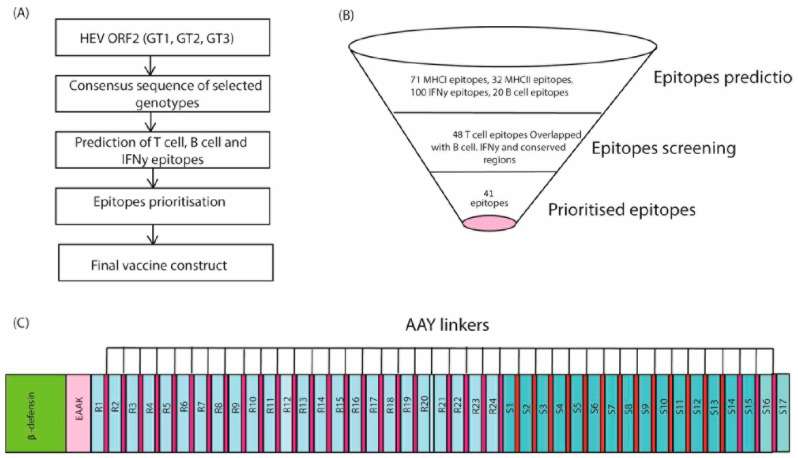
Predicted 3D structure and validation of multi-epitope vaccine constructs (**A**) Vu; universal vaccine construct (**B**) Ramachandran plot showed that 88.3% (Vu) of the proposed vaccine constructs residues were present in favored regions. (**C**) ProSA-web results indicated a Z-score of −0.86.

The present study was divided into six key parts (Figure 2)

1. Selection of conserved regions among the ORF2 region of the selected HEV genotypes (GT1, 3, and 4);
2. Screening of experimentally-validated antigenic regions of conserved HEV ORF2 regions through data mining;
3. Prediction of T cell, IFN-γ and B cell epitopes;
4. Screening of overlapping T cell, IFN-γ and B cell, epitopes and experimentally-validated ORF2 antigenic regions;
5. The fusion of immunogenic, antigenic epitopes with appropriate linkers and adjuvant;
6. Validation of the proposed vaccine construct (Vu) structure and interactions.

### 2.1. Sequence Collection

We retrieved 53 ORF2 sequences of HEV genotype 1, 133 of genotype 3, and 105 ORF2 sequences from genotype 4 from the GeneBank. All leading genotypes of the HEV (1, 3, and 4) were included in the study. 

### 2.2. Consensus Sequence

The sequences of the HEV ORF2 region were aligned using BioEdit and CLC Workbench 7 [3] to produce the consensus sequence of each HEV genotype, 1, 3, and 4. The consensus sequences were then aligned to obtain a global consensus sequence. 

### 2.3. Retrieval of Antigenic Regions by Data Mining

An extensive literature search was performed to screen out all experimentally validated epitopes or antigenic regions in HEV ORF2. Studies evaluating the role of different antigenic regions of ORF2 were considered, and each antigenic region was detailed for further evaluation of selected T cell epitopes. 

### 2.4. Prediction of Epitopes

MHC I, and MHC II-based T cell epitopes, B cell epitopes, and IFN-γ inducing epitopes were predicted by using the consensus sequence of selected genotypes. HLApred [11] was used to predict the MHC I and II T cell epitopes. For the prediction of B cell epitopes the online immune epitope database, IEDB (http://tools.iedb.org/bcell/ (last accessed on 22 July 2022)), was utilized. We applied highly restrictive thresholds and screen out epitope sequences which overlapped with experimentally validated epitopes in the IEDB. Furthermore, for the prediction of IFN-γ epitopes, the IFNepitope (http://crdd.osdd.net/raghava/ifnepitope/ (last accessed on 22 July 2022)) server was used. This server predicts the results of the amino acid composition of experimentally validated IFN-gamma-inducing epitopes.

### 2.5. Selection of Epitopes

All T cell epitopes overlapping with the prophesied epitopes of IFN-γ and B cell were prioritized. The selection was extended to prioritize overlapping epitopes lying within conserved regions and experimentally validated antigenic regions. Screened epitopes were then again checked for their antigenicity using VaxiJen [12] with a cutoff score of ≥0.5. 

### 2.6. Comparative Analysis with Human Proteins

BLASTp [13] was used to screen nonhuman homolog epitopes from the selected epitopes. These epitopes were selected for further consideration.

### 2.7. Construction of the Multi-Epitope Vaccine

In this study, we adopted strict criteria for the prediction of a multi-epitope vaccine construct against HEV. Linkers, such as AAY, GPGPG, and GGGGGS were added between prioritized epitopes to achieve better expression levels, solubility, and the perfect folding of multi-epitope structures. Each linker was either added individually adjacent to each epitope or in combination with other linkers. Their antigenicity and allergenicity were further verified with different combinations by VaxiJen [12] and AllergenPro [9]. Finally, the best linker combination was selected, and adjuvant β-defensin was conducted at its N-terminal along with the EAAAK linker. 

### 2.8. Prediction of the 2D and 3D Structure

PSIPRED [10] software was used for the prediction of alpha, beta-helix, and coil structures of vaccine constructs. Its accuracy is 81.6%. The I-TASSER server was used to predict the tertiary structure of the final vaccine construct. It employs a hierarchical approach to predict protein structure. It is one of the best protein structure prediction software packages [14]. For refinement of the 3D structure, the online tool GalaxyRefine and ModRefiner [15] were used. Further validation of the 3D structure was performed by generating the Ramachandran plot (http://mordred.bioc.cam.ac.uk/~rapper/rampage.php (accessed on 15 August 2022).

### 2.9. Molecular Dynamics Simulation and Molecular Docking

Molecular dynamic simulations were run against predicted vaccine constructs using GROMACS at 10 ns for 50,000 steps. This program provides a realistic cellular environment to the given biological model. In a dodecahedron box, the vaccine construct was enclosed, and an Optimized Potential for Liquid Simulation force-field (OPLSA) was employed. Spc216.gro water was used as a solvent to simulate all proteins. The overall charge on proteins was calculated and neutralized by addition of Na^+^ and Cl^−^ ions using GROMACS tool Genion [16]. The energy of each protein structure was minimized at 50,000 steps. Later, the protein was equilibrated using NVT isothermal-isochoric and NPT ensemble at 50,000 steps. The pressure, temperature, and density of each vaccine construct was analyzed using the generated graph. Finally, MD simulation at 500,000 steps was employed, and a stable frame from the trajectory was picked using VMD. Then, molecular docking of vaccine constructs with TLR3 (1 ziw) was performed based on the observation that the patients with higher expressions of TLR3 were able to limit HEV and recover. The patients showing lower expressions of TLR3 developed acute liver failure (ALF) [17]. For docking, HADDOCK was used [18]. Furthermore, to predict interacting residues involved in the molecular interactions of the designed vaccine construct and the receptor (TLR3), the online database PDBsum was utilized [19].

## 3. Results

### 3.1. Identification of Conserved Regions in HEV ORF2

Conserved regions of virus genomes are considered one of the potential regions for vaccine design. Therefore, we used a conserved region HEV genome to design a HEV universal vaccine construct (Vu) against common genotypes (GT 1, 3, 4) and performed conservation analysis. CLC workbench [20] and BioEdit [21] software have predicted 17 conserved regions in the consensus sequence (Supplementary Table S1).

### 3.2. Prediction of Antigenic Epitopes

The consensus sequence of selected genotypes (1, 3, 4) was used to predict 9-mer T cell (MHC class I and class II) epitopes. In this study, the T cells with binding affinity to the maximum number of human alleles were prioritized. A total of 71 MHCI epitopes and 32 MHCII unique epitopes against all major alleles were identified. IFNepitopes and IEDB servers predicted 100 IFN-y epitopes and 20 mer B cell epitopes. Furthermore, 48 MHC-I and II epitopes overlapped with the IFN-y and B cell epitopes, and conserved regions were prioritized. BLASTp [13] indicates that not a single epitope in our study is homologous to the human protein. We used VaxiJen tool [12] to check the antigenicity of the prioritized epitopes. Epitopes with high antigenicity were selected for further analysis.

### 3.3. Screening of Experimentally Validated Antigenic Regions through Data Mining

In this study, all possible antigenic regions and epitopes of HEV ORF2 regions were explored and validated with experiments. Different words were used for exploring antigenic regions of HEV (Supplementary Table S2). By using these words, we found many antigenic regions in ORF2. HEV ORF2 is a 660 amino acid (aa) protein, and the E2 domain (459–606 aa) is considered one of the most antigenic regions of ORF2 [22]. The Hecolin vaccine candidate designed by GlaxoSmithKline (GSK) encompasses 368–606 aa of ORF2 region and is also known to be immunogenic [23]. Another study revealed that 30-mer synthetic peptides isolated from domains 1 (12–147), 4 (403–465), and 6 (592–660) were immuno-reacted with greater than 70% of anti-HEV-positive serum samples [24]. Another study states that the sequences from aa 394–457 of the ORF2 contribute to the development of highly immunogenic epitopes [25]. Based on observation, we believed that all T and B cell epitopes predicted in this study against the ORF2 region should be present in these immunogenic regions (Table 1). Overlapping epitope sequences were merged if they did not affect the overall antigenicity of the epitope. Their antigenicity was cross-checked with the VaxiJen server [12]. Based on the strict screening approach, the prioritized epitopes have the following features: (1) Maximum binding affinity to a large number of human alleles; (2) present in the conserved region and colocalized with predicted epitopes (IFN-γ and B cell); and (3) highly antigenic and nonhuman homologs. Based on these filters, 41 epitopes from the consensus sequence of GT 1, 3, 4 were prioritized.

### 3.4. Designing of the Vaccine Construct (Vu)

All prioritized 41 epitopes were linked by flexible linkers (AAY, GPGPG, GSSSSS). Each linker was added between the epitopes, their allergenicity and antigenicity were further checked with VaxiJen and AllergenPro. We also checked different combinations of linkers between MHCI and MHCII epitopes for their antigenicity and allergenicity (Figure 2). We found that Vu was more antigenic with the AAY linker when compared with other linker combinations. Finally, at the N-terminal of the vaccine construct, adjuvant β-defensin (45 aa) (GIINTLQKYYCRVRGGRCAVLSCLP-KEEQIGKCSTRGRKCCRRKK) was added with the help of EAAK linker (Figure 2). 

### 3.5. Prediction and Validation of the Vaccine Structure

**Figure 3:**
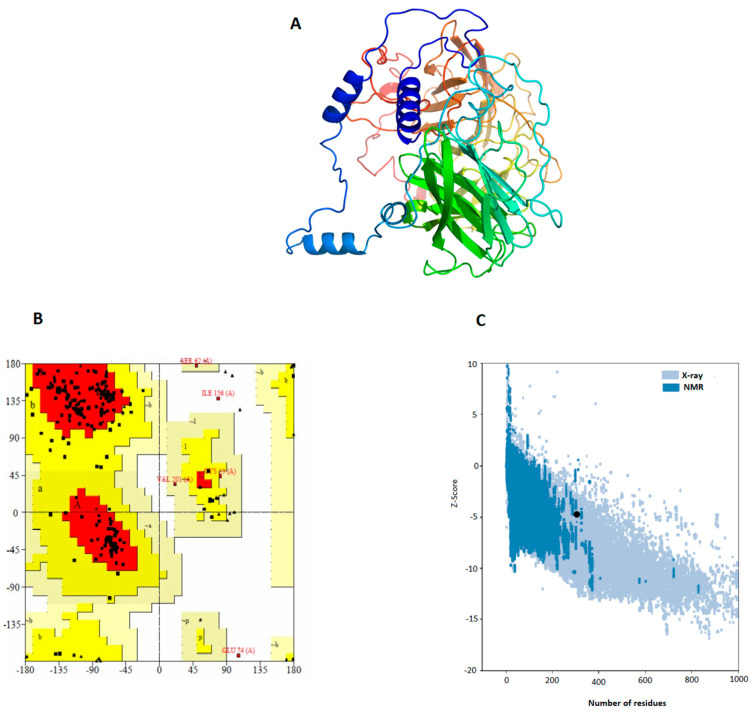
Multi-epitope vaccine constructs predicted 3D organization and their validation (**A**) Vu; universal vaccine construct (**B**) Ramachandran plot showed that 88.3% (Vu) of the proposed vaccine construct residues were present in favored regions. (**C**) ProSA-web results indicated a Z-score of −0.86.

PSIPRED was used to predict the 2D structure of the Vu [15]. It shows that the vac-cine construct has alpha-helices, beta-sheets, and beta-turn. Three-dimensional structures of final vaccine constructs were predicted with I-TASSER. Based on the threading templates, it predicts five tertiary structure models of the given protein. The C-score of the predicted structures is in the range of −5 and 2, while higher values represent higher confidence. The vaccine construct was refined using GalaxyRefine and ModRefiner server. Validation of the vaccine constructs was performed by the Ramachandran plot and ProSA analysis. The secondary structure and Ramachandran plot validation of the vaccine construct (Vu) have been given in Figure 3. We found that in Vu 89.3% of the region lies in the favored region, 9.7% in the allowed region, and 1% in the outlier region (Figure 3). The present results confirm that most of the amino acid phi-psi distributions are constant with a right-handed α-helix and the models were stable and reliable. ProSA-web results showed a Z-score of −0.86, which represents a high quality of the given structure.

### 3.6. Molecular Simulations and Docking Analysis with TLR3

**Figure 4:**
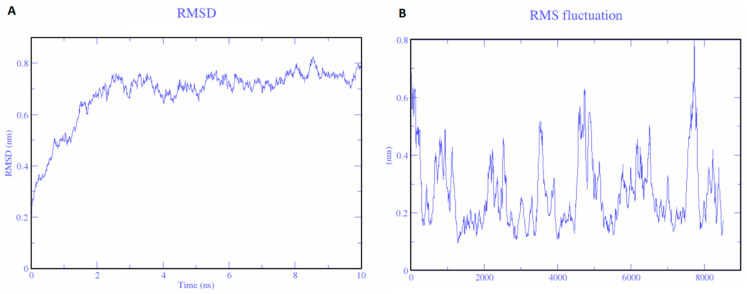
Molecular dynamics simulations of the vaccine candidates. (**A**) RMSD; RMSD levels off to 0.5 (Vu), this shows the stability of the vaccine construct. (**B**) RMSF; RMSF-Root Mean Square Fluctuation plot, heights show the high-flexibility regions.

**Figure 5:**
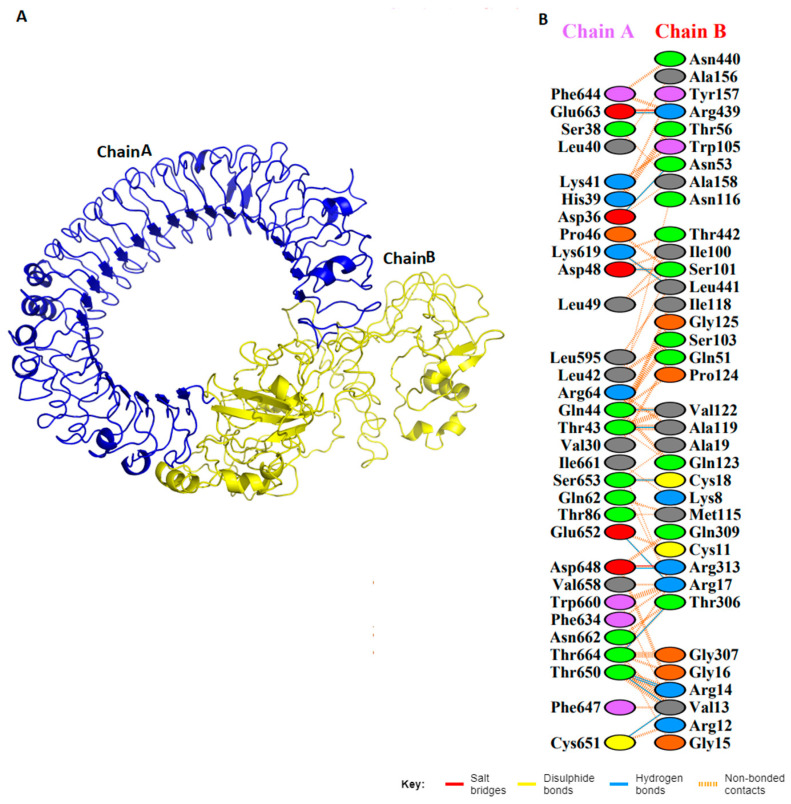
(**A**) Vu-TLR3–docked complex of the vaccine construct: we acquired these figures by performing molecular docking between TLR-3 and the vaccine. The blue shows TLR-3 and yellow represents the vaccine construct. (**B**) Chain A and B interactions.

The vaccine construct showed peculiar characteristics. The charge on the protein was +10. Therefore, ten chloride ions were added by substituting water molecules at atoms 23212, 49258, 40630, 141907, 144796, 31747, 57016, 14941, 38455, and 135937. The energy minimization was achieved at 50,000 steps. The potential energy was −2.714397e + 6, and the average potential energy of the system was −2.04264e + 06, with a drift of −255,072 KJ/mol. The temperature of Vu was maintained at around 300K. RMSD of the protein is shown in Figure 4. Stable conformation of the protein was at the 101st coordinate. Hence, it was selected for further analysis. After MD, molecular docking of Vu was performed with TLR3 using the HADDOCK server [27]. HADDOCK predicts various models, and the highest 10 clusters were nominated. While the topmost cluster was considered the reliable model as it possesses the lowest HADDOCK energy score, i.e., −5.8 ± 58.2. The reliability of each model was determined by its Z-score. The higher negative Z-score signifies a better structure. Good interactions between the vaccine construct (Vu) and TLR3 were determined. TLR3 is shown in blue and vaccine constructs are shown in yellow (Figure 5). To further analyze these interactions, a schematic illustration of interactions was obtained through PDBsum [21]. We observed that Vu established 21 hydrogen bonds with TLR3 with the distance of 2–3 Å representing strong interactions. 

## 4. Discussion

HEV is a fecal-orally transmitted foodborne pathogen that causes acute hepatitis in humans and emerged as an important public health concern in the world [28]. HEV has a significant risk of severe disease outcomes in pregnant women and those diagnosed with chronic liver disease [29]. There are many immune-related concerns with traditional vaccines, including pathogens with complex lifecycle and antigenic variations, the need for personalized vaccinations, and concerns for live attenuated vaccines and the initiation of non-specific immune responses that may cause autoimmunity and vaccine allergy. Recent studies demonstrated a potential role of HEV in developing autoimmune diseases with increasing rates of autoimmune hepatitis (AIH) [30]. In addition, complete ORF2 antigens in HEV-infected patients reduced the protective efficacy after vaccination. Further immunological characterization of ORF2 and its kinetics in blood is of great importance [31]. These concerns have forced immunologists to design a better HEV vaccine approach that will consider these challenges. Immunoinformatic methods can be used to assign functions to uncharacterized genes. This can be a better approach to design vaccines against the pathogens affecting immunocompromised patients, especially pregnant women who are mainly not involved or do not participate in clinical trials. It has been reported that immuno-bioinformatic approaches are best for screening epitopes to design candidate vaccines, particularly the HEV vaccine [32]. In the present study we have used the immunoinformatic approaches to critically analyze the immunogenic ORF2 region of HEV common genotypes to prioritize the immunogenic epitopes with higher affinity and immunogenicity. The ORF2 of HEV has shown the potential to be a vaccine candidate in various experimental studies [26,33]. This strategy was adopted to recognize the immunogenic epitopes against each genotype located within the conserved and experimentally validated regions, confirming the importance of our study.

The effective vaccines of HEV should be proficient in activating both cellular and humoral immune responses in humans. This study proposes a set of immunogenic epitopes that could produce both T cell and B cell immune responses. This could help to develop an epitope-based vaccine against HEV infection. Our proposed methodology in finding immunogenic epitopes involves the identification of overlapping regions of T cell, IFN-y, and B cell epitopes from ORF2 of HEV, mainly at those spots where these epitopes are present in reported HEV antigenic regions. Based on the results, we have shortlisted 37 epitopes of the consensus sequence. These epitopes have T cell epitopes overlapping B cell. IFNy epitopes thus can stimulate T cell, B cell, and IFNy responses, simultaneously. IFN-gamma is the signature cytokine of adaptive and innate adaptive immune systems, consequently epitopes having the ability to induce interferon gamma could increase the immunogenicity of any vaccine to combat the pathogen [34]. IFNγ is an essential element in the regulation of the host immune response against viral and intracellular bacterial pathogens. Thus, identifying the epitopes having the ability to activate Type 1 T helper cells, CD8^+^ cytotoxic T lymphocytes will be helpful in developing vaccines with a more specific immune response. The selected 41 epitopes were revealed to be non-toxic, non-allergen, and impotent of initiating autoimmune responses, which represents their potential as effective vaccine candidates. The screened epitopes were further connected with different linkers GPGPG, AAY, GGGGGS, and the best combination with high antigenicity was selected as a vaccine candidate. The EAAAK linker, a helical protein, was also added between β-defensin and the combined epitopes. The vaccine candidate was predicted by I-TASSER and was refined by GalaxyRefine and ModRefine. Moreover, the structural validation highlights the high structural integrity of the vaccine construct by showing the maximum percentage (89.3%) of residues in the favorable zone. The Vu was also shown to be a stable entity by predicted RMSD. 

It has been revealed by previous studies that a highly glycosylated vaccine has low antigenicity and affords significantly less protection than a low glycosylated protein [34]. Different studies based on animal models revealed that the glycosylation of key vaccine epitopes might significantly affect vaccine properties [34]. However, in other studies it is revealed that the glycosylation of epitopes may enhance the immunogenicity of the viral proteins. T Cell populations that recognize glycoepitopes presented by MHC-I and MHC-II have been identified in previous studies confirming the importance of glycosylated epitopes. Glycosylation is required for achieving the conformational integrity of various epitopes, to enhance its immunogenicity and to further prevent its proteolytic cleavage [34,35]. Glycosylation of viral envelope proteins influences the immunogenicity and the sensitivity of the virus to neutralizing antibodies [34]. The ORF2 protein sequence contains three conserved glycosylation sites at the position of 137, 310 and 562. Four of the conserved predicted epitopes (R7, r3, r9, R23) for the development of the vaccine construct in the present study also possess glycosylation sites. Presence of these glycosylated epitopes in the designed vaccine construct confirms the immunogenicity and sensitivity of proposed the vaccine construct.

Epitope-based vaccines can only produce a steady immune response if it is effectively recognized by the target immune cell receptors [14]. Toll-like receptors (TLRs) have a strong ability to detect the conserved pathogen-associated molecular patterns (PAMPs) of various microorganisms and the activation of the immune response [15]. The therapeutic potential of TLR3 to limit HEV infection has already been reported [16]. TLR3 is involved in the recognition of viral structural proteins and the production of inflammatory cytokines [17]. It triggers downstream, signaling cascades of IRF3 and NF-κB, resulting in the production of interferons and inflammatory cytokines. In HEV-infected pregnant women, there are high expression levels of TLR3 and IFN-γ [16]. Overexpression of TLR3 has been observed in cell culture models resulting in inhibition of HEV replication [19]. Finally, to investigate the potential immune association between TLR3 and the vaccine construct, molecular docking analysis was performed, which showed strong interactions between the two. We observed that our multi-epitope vaccine construct created several intermolecular interactions, such as hydrogen bonding with human TLR3. Stable interactions between the vaccine construct and the TLR3 receptor have been observed in RMSD analysis. These results suggest that these 41 epitopes are highly conserved with efficient expression potential. Therefore, the development of HEV vaccines using these 41 immunogenic epitopes could stimulate both humoral and cellular immune responses in humans.

## 5. Conclusions

With the help of various immunoinformatic approaches, we have designed a conserved universal epitope vaccine against major HEV genotypes. This vaccine may have the ability to stimulate both humoral and cellular immune responses and can tackle the emerging RNA mutations of HEV. The potential immune association between TLR3 and vaccine construct was also observed, presenting it as an ideal candidate against the HEV vaccine. However, further experimental validations are still required to confirm these findings.

## Figures and Tables

**Table 1:** Finalized epitopes for the HEV vaccine construct.

MHCI Epitopes	Name	References	MHCII	Name	References
	All genotypes (GT1, GT3, GT4)				
^23^GQPSGRRRG^31^	R1	[24]	^8^LLLLVLLPM^16^	r1	[24]
^134^RQYNLSTSP^142^	R2	[24]	^11^LVLLPMLP^19^	r2	[24]
^136^NLSTSPLT^144^	R3	[24]	^132^RRQYNLST^140^	r3	[24]
^162^PLLPLQDGT^170^	R4	[24]	^187^VVRATIRYR^195^	r4	[24]
^170^NTHIMATEEASNY^183^	R5	[24]	^192^IRYRPLVPN^200^	r5	[24]
^199^PNAVGGYAISISFWPQTTTT^218^	R6	[24]	^202^VGGYAISIS^210^	r6	[24]
^136^YNLSTSPLT^144^	R7	[24]	^258^WRSVETSGV^266^	r7	[24]
^162^PLLPLQDGT^170^	R8	[24]	^275^LVMLCIHGS^283^	r8	[24]
^170^NTHIMATEEASNY^183^	R9	[24]	^308^FRNLTPGNT^316^	r9	[24]
^199^PNAVGGYAISISFWPQTTTT^218^	R10	[24]	^349^FMKDLHFTG^357^	r10	[24]
^219^PTSVDMNSITSTDV^232^	R11	[24]	^353^LHFTGTNGV^361^	r11	[24]
^236^VQPGIASEL^244^	R12	[24]	^373^NLADTLLG^381^	r12	[23,24,26]
^251^LHYRNQGWRSVET^263^	R13	[24]	^396^FYSRPVVSA^404^	r13	[23,24,26]
^267^AEEEATSGLV^276^	R14	[24]	^465^LRANDVLWL^473^	r14	[23,24,26]
^303^ALELEFRNLTPGNTNT^218^	R15	[24]	^500^FVNVATGAQ^509^	r15	[23,24,26]
^339^AELTTTAATR^348^	R16	[24]	^519^VTLDGRPLT^527^	r16	[23,24,26]
^370^LTLFNLADT^378^	R17	[23,24,26]	^536^FFVLPLRGKLSFWE^538^	r17	[23,24,26]
^403^SANGEPTVKLY^413^	R18	[23,24,26]			
^413^YTSVENAQQ^421^	R19	[23,24,26]			
^440^IQDYDNQHEQ^449^	R20	[23,24,26]			
^465^LRANDVLWL^473^	R21	[23,24,26]			
^467^NDVLWLSLTAAEYDQ^482^	R22	[23,24,26]			
^544^KLSFWEAGTTKAGYPYNYNTTA^565^	R23	[23,24,26]			
^645^AELQRLKMK^653^	R24	[23,24,26]			

## Data Availability

The datasets generated and analyzed during the current study are not publicly available due to the risk of compromising the individual privacy of participants, but are available from the corresponding author on reasonable request.

## Supplementary Materials

The following supporting information can be downloaded at: https://www.mdpi.com/article/10.3390/vaccines11030710/s1, Table S1: Conserved regions of ORF2: Seventeen conserved regions in ORF2 of HEV; Table S2: Search theme: Words for data mining antigenic regions of HEV.
