# Supplementary material for: An In Silico Deep Learning Approach to Multi-Epitope Vaccine Design: A Hepatitis E Virus Case Study
Source: Vaccines (Basel). 2023 Mar 22;11(3):710. doi: 10.3390/vaccines11030710 (PMC10059858; doi:10.3390/vaccines11030710)
Supplement: Supplementary file 1 [file vaccines-11-00710-s001.zip › vaccines-2051864-supplementary.pdf]

Supplementary Table S1: Conserved regions of ORF2

Seventeen conserved regions in ORF2 of HEV

GQPSGRRRGRRSGG	22
GGGFWGDRVDSQPFA	37
PYIHPTNPFA	54
RPLGSAWRDQ	80
PVPDVDSRGAILRRQYNLSTSPLTS	120
GTNLVLYAAPL	149
PLLPLQDGTNTHIMATEASNYAQYRV	161
ATIRYRPLVPNAVGGYAISISFWPQTTTTPTSVDMNSITSTDVRILVQPGIAS ELVIPSERLHYRNQGWRSVET	188
GVAEEEATSGLVMLCIHGSPVNSYTNTPYTGALGLLDFALELEFRNLTPGN TNTRVSRY	264
DGTAELTTTAATRFMKDL	335
GIALTLFNLADTLLGGLPTELISSAGGQLFYSRPVVSANGEPTVKLYTSVEN AQQDKGI	366
RVVIQDYDNQHEQDRPTPSPAPSRPFSVLRANDVLWLSLTAAEYDQ	436
KVTLDGRPL	517
TIQQYSKTF	527
VLPLRGKLSFWEAGTTKAGYPYNYNTTASDQ	537
DYPARAHTFDDFCPECR	614
AELQRLKMKVGKTRE	644

Supplementary Table S2: Search theme: Words for data mining antigenic regions of HEV

HEV antigenic epitope
HEV epitopes
Immunogenic regions of HEV
Vaccine regions of HEV
Experimentally validated epitopes of HEV
Position of epitopes in HEV
